# Changes in anterior segment parameters after laser peripheral iridotomy in patients with primary angle closure suspect

**DOI:** 10.3389/fmed.2026.1784392

**Published:** 2026-05-08

**Authors:** Qi Feng, Zenghui Chen, Daliang Wang, Guomei Yuan, Jiang Shen, Li Zhang, Man Luo, Wei Chen, Surong Luo

**Affiliations:** 1 Department of Ophthalmology, Shaoxing People's Hospital, Zhejiang University, Shaoxing, China; 2 School of Medicine, Shaoxing University, Shaoxing, Zhejiang, China

**Keywords:** anterior chamber angle, anterior chamber depth, anterior chamber volume, laser peripheral iridotomy, primary angle closure suspect

## Abstract

**Objective:**

To evaluate changes in anterior segment parameters before and after laser peripheral iridotomy (LPI) in patients with primary angle closure suspect (PACS), analyze the role of anterior chamber anatomical changes in the development of primary angle closure glaucoma (PACG), and assess the clinical value of LPI in PACS.

**Method:**

A total of 42 patients (42 eyes) diagnosed with PACS and treated with LPI at the Ophthalmology Department of Shaoxing People’s Hospital between January 2023 and April 2025 were included. Anterior chamber volume (ACV), anterior chamber depth (ACD), and anterior chamber angle (ACA) were measured using the Sirius anterior segment analysis system before surgery and at 1 week and 3 months postoperatively. Visual acuity, intraocular pressure, and corneal endothelial cell density were also assessed. Comparisons among time points were performed using SPSS 25.0, with *p* ≤ 0.05 considered statistically significant.

**Result:**

This study included 7 males and 35 females aged 41–84 years (mean age, 62.62 ± 9.50 years), with a mean axial length of 22.09 ± 0.82 mm. There were no significant differences in visual acuity or intraocular pressure across the three time points (*p* > 0.05). Corneal endothelial cell density showed no significant change between preoperative and 1-week postoperative measurements (*p* > 0.05). In contrast, ACD increased significantly from 1.87 ± 0.04 mm preoperatively to 2.02 ± 0.05 mm at 1 week and 2.03 ± 0.05 mm at 3 months postoperatively (*p* < 0.05). ACV increased significantly from 113.48 ± 3.18 mm3 preoperatively to 123.06 ± 2.54 mm3 at 1 week and 122.50 ± 2.18 mm3 at 3 months (*p* < 0.05), and ACA widened significantly from 30.26 ± 0.65° to 33.90 ± 0.79° and 33.34 ± 0.79°, respectively (*p* < 0.05).

**Conclusion:**

LPI in patients with PACS is safe, as it does not adversely affect visual acuity, intraocular pressure, or corneal endothelial cell density. The procedure significantly deepens the anterior chamber, increases ACV, and widens the ACA, supporting its role in improving anterior segment anatomy and potentially delaying progression to primary angle closure and PACG.

## 1 Introduction

Glaucoma is a group of diseases characterized by optic nerve damage and visual field defects, and is the second leading cause of blindness worldwide. Epidemiological studies have shown that primary angle closure glaucoma (PACG) has a high incidence among Asian populations, estimated at 1.09%. By 2040, the number of glaucoma patients worldwide is expected to reach 111.8 million (1). In 2002, Foster (2) classified the development of PACG into three stages based on the International Society for Geographical and Epidemiologic Ophthalmology system: primary angle closure suspect (PACS), primary angle closure (PAC), and primary angle closure glaucoma (PACG). Among these, PACS carries a high risk of progressing to PAC and PACG. The pathophysiology of PACG is pupillary block, which leads to the closure of the angle structures responsible for aqueous humor drainage, thereby disrupting the balance between aqueous humor production and outflow. Anterior chamber angle (ACA) is closely associated with pupillary block. Therefore, evaluation of the ACA is essential for diagnosing and assessing the efficacy of glaucoma-related interventions.

For the early intervention of angle-closure glaucoma, laser peripheral iridotomy (LPI) is widely applied in clinical practice. LPI is an important treatment for pupillary block–type glaucoma, as it can prevent the progression from PAC to PACG and reduce the risk of recurrent acute attacks (3, 4).

In this study, we aimed to evaluate patients with PACS who underwent LPI. Using the Sirius anterior segment analysis system, we measured anterior chamber depth (ACD), anterior chamber volume (ACV), and ACA before and at multiple time points after LPI. The study confirmed that LPI can improve anterior chamber angle and anatomy in patients with PACS and clarified the clinical significance of early LPI intervention, providing a basis for preventing progression to PAC and PACG.

## 2 Patients and methods

### 2.1 Study participants

Patients who visited the Ophthalmology Department of Shaoxing People’s Hospital between January 2023 and April 2025, and were diagnosed with PACS based on Ultrasound Biomicroscopy (UBM) and gonioscopy, were included in this study. A total of 42 patients (42 eyes) were included. All patients provided written informed consent, and LPI was performed on the affected eyes. This study was approved by the Ethics Committee of Shaoxing People’s Hospital (approval number: 2023 Scientific Research Project No. 056-Y-01).

Inclusion criteria were: no acute PACG attack, no optic nerve head changes, no corneal visual field damage, intraocular pressure (IOP) < 21 mm Hg, no peripheral anterior synechiae, and no history of ophthalmic surgery. Affected eyes had narrow anterior chambers, defined as at least 180° of the angle being nonvisible on dynamic gonioscopy. Exclusion criteria included severe systemic diseases affecting follow-up, considerable corneal opacities preventing laser treatment, or acute PAC attack with iris adhesion, local iris atrophy, or glaucomatous spots.

Detailed information on patient age, sex, and concurrent systemic diseases was recorded. All patients underwent comprehensive ophthalmic examinations before LPI, including best-corrected visual acuity (BCVA), IOP, slit-lamp microscopy, corneal endothelial cell count, Humphrey visual field test, optical coherence tomography, UBM, and anterior segment analysis using the system. ACD, ACV, and ACA were measured before LPI, 1 week after, and 3 months postoperatively. The same examiner conducted all examinations.

Anterior segment parameters were measured using the Sirius system (CSO, Italy). Patients were seated with the lower jaw on the chin rest and forehead against the forehead strip, instructed to look straight ahead with the affected eye fully open. The patient focused on a blue light target while the Scheimpflug camera captured 25 images and one Placido disc image within 2 s. Images with quality > 90% were selected, and the system automatically generated the ACD map, from which ACD, ACV, and ACA were recorded.

### 2.2 Surgical method

The lead surgeon performed all LPI procedures. Forty minutes prior to surgery, pilocarpine nitrate eye drops were instilled four times at 10-min intervals to constrict the pupil. Surface anesthesia was achieved with a single instillation of 0.5% hydrochloric acetylprocaine eye drops. The Abraham lens was placed on the cornea, and the incision site was selected at the outer one-third of the peripheral iris, typically at the 1–2 o’clock or 10–11 o’clock positions, avoiding the corneal senile ring and vascular tufts. An Nd: YAG laser (Ellex Super Q, Australia) was used, with energy starting at 4.0 mJ and gradually increased until the iris was perforated, creating a 0.3–0.5 mm hole for direct visualization of the posterior chamber. If the iris was not perforated, 0.1% fluorometholone eye drops were instilled for 2–3 days before repeating the procedure. The procedure was conducted in a dark room. Postoperatively, patients received compound tobramycin eye drops for 3 days. IOP was measured 1 h after surgery, and appropriate treatment was provided as needed for elevated pressure or bleeding.

### 2.3 Statistical analysis

Statistical analyses was performed using SPSS 25.0. Mauchly’s test of sphericity was applied to ACD, ACV, and ACA measured at three time points (preoperatively, 1 week postoperatively, and 3 months postoperatively). A *p*-value > 0.05 was taken to be indicative of a lack of correlation among values obtained at the different time points. Data conforming to a normal distribution were analyzed using a one-way analysis of variance (ANOVA). Data for which a Mauchly test *p*-value < 0.05 was obtained, indicating a correlation among the ACD, ACV, and ACA values at different time points. If the data conformed to a normal distribution, repeated-measures ANOVA was employed, followed by pairwise comparisons of ACD, ACV, and ACA at the different time points using Tukey’s honestly significant difference method. A *p*-value ≤ 0.05 was considered statistically significant.

## 3 Results

### 3.1 Patient demographics and clinical characteristics

A total of 42 patients (7 males, 35 females, 42 eyes) diagnosed with PACS were included in this study. Of the enrolled patients, 28 underwent bilateral LPI; for these cases, the left eye was uniformly selected as the study eye. The remaining 14 patients received unilateral LPI. Among them, 13 patients had a family history of glaucoma; 40 eyes were in the clinical pre-stage of acute primary angle-closure. Patient age ranged from 41 to 84 years, with a mean age of 62.62 ± 9.50 years. The mean axial length of the included eyes was 22.12 ± 0.82 mm (Table 1).

**Table 1:** Patient demographics and clinical characteristics.

Characteristics	Mean ± SD	Range
Sex (Male/Female)	7/35	16.7%:83.3%
Right eye/Left eye	14/28	33.3%:66.7%
Family history of glaucoma (yes/no)	13/29	31%; 69%
Age (Mean ± SD)	62.62 ± 9.50	41–84
Axial lengths (mm)	22.09 ± 0.82	20.22 ~ 23.76
Types of glaucoma		
The clinical pre-stage of acute primary angle-closure (*n*)	40	95.2%
Shallow anterior chamber and narrow angles (*n*)	2	4.8%

### 3.2 BCVA, IOP, and corneal endothelial cell density (ECD)

Among the 42 patients (42 eyes), BCVA before surgery and at 1 week and 3 months postoperatively was 0.50 ± 0.27, 0.50 ± 0.27, and 0.52 ± 0.26, respectively. No statistically significant difference in visual acuity was observed across the three time points (*p* = 0.356). IOP before surgery and at 1 week and 3 months postoperatively was 14.24 ± 3.45, 13.66 ± 2.68, and 13.74 ± 2.69 mmHg, respectively, with no statistically significant difference among time points (*p* = 0.543). ECD before surgery and at 1 week postoperatively was 2748.12 ± 298.13 and 2737.05 ± 294.45 cells/mm^2^, respectively. No statistically significant difference was detected between these two measurements (*p* = 0.130; Table 2).

**Table 2:** BCVA, IOP, and ECD at different time points before and after LPI.

	BCVA	IOP (mmHg)	ECD (mm/^2^)
Before LPI	0.50 ± 0.27	14.24 ± 3.45	2748.12 ± 298.13
1 week after LPI	0.52 ± 0.27	13.66 ± 2.68	2737.05 ± 294.45
3 months after LPI	0.52 ± 0.26	13.74 ± 2.69	
*p*	0.356	0.543	0.130

LPI, laser peripheral iridotomy; IOP, intraocular pressure; ECD, corneal endothelial cell density.

These results indicate that BCVA did not decline after LPI, IOP remained stable, and ECD was not reduced, demonstrating the safety of LPI in patients with PACS.

### 3.3 ACD, ACV, and ACA

In the 42 patients (42 eyes), ACD was 1.87 ± 0.04 mm preoperatively, 2.02 ± 0.05 mm at 1 week postoperatively, and 2.03 ± 0.05 mm at 3 months postoperatively. A statistically significant difference in ACD was observed across time points (*p* < 0.001). ACV increased from 113.48 ± 3.18 mm^3^ preoperatively to 123.06 ± 2.54 mm^3^ at 1 week and 122.50 ± 2.18 mm^3^ at 3 months postoperatively, with a statistically significant difference among the time points (*p* = 0.024). ACA widened from 30.26 ± 0.65° preoperatively to 33.90 ± 0.79° at 1 week and 33.34 ± 0.79° at 3 months postoperatively. This change was also statistically significant (*p* < 0.001; Table 3). Pairwise comparisons of anterior segment parameters before and after LPI are shown in Table 4.

**Table 3:** Comparison of anterior segment parameters across time points within groups before and after LPI.

	ACD (mm)	ACV (mm^3^)	ACA (°)
Before LPI	1.87 ± 0.04	113.48 ± 3.180	30.26 ± 0.65
1 week after LPI	2.02 ± 0.05	123.06 ± 2.54	33.90 ± 0.79
3 months after LPI	2.03 ± 0.05	122.50 ± 2.18	33.34 ± 0.79
*p*	0.000*	0.000*	0.000*

^*^p < 0.01. LPI, laser peripheral iridotomy; ACD, anterior chamber depth; ACV, anterior chamber volume; ACA, anterior chamber angle.

**Table 4:** Pairwise comparison of anterior segment parameters before and after LPI.

		Average value	Mean variation	*p*
ACD	Before and 1 week after LPI	−0.154	0.062	0.038*
	Before and 3 months after LPI	−0.164	0.062	0.024*
	1 week and 3 months after LPI	−0.011	0.062	0.984
ACV	Before and 1 week after LPI	−9.578	3.768	0.033*
	Before and 3 months after LPI	−9.013	3.768	0.048*
	1 week and 3 months after LPI	0.565	3.768	0.988
ACA	Before and 1 week after LPI	−3.645	1.053	0.002**
	Before and 3 months after LPI	−3.083	1.053	0.011*
	1 week and 3 months after LPI	0.562	1.053	0.855

*p < 0.05; **p < 0.01. LPI, laser peripheral iridotomy; ACD, anterior chamber depth; ACV, anterior chamber volume; ACA, anterior chamber angle.

Overall, ACD increased, ACV enlarged, and ACA widened after LPI in patients with PACS. No statistically significant differences were observed between measurements at 1 week and 3 months postoperatively, indicating that these parameters stabilized from 1 week onward after surgery.

## 4 Discussion

PACG comprises a group of disorders characterized by acute or chronic elevation of IOP caused by primary angle closure, leading to optic disc changes and visual field defects (5, 6). PACG has a particularly high prevalence in Asian populations, with PACS reported to affect 10.4% of individuals in China (7). It is estimated that more than 28 million people in China have PACS, 9 million have PAC, and 4.5 million have PACG (8). Thomas (9) reported that, without intervention, 22% of PACS cases may progress to PAC, and 28.5% of PAC cases may further progress to PACG, highlighting the importance of early identification and intervention.

Glaucoma-induced damage to the optic nerve is irreversible. Therefore, screening and early identification of high-risk individuals are crucial for preventing disability and blindness. A study by Grimes et al. (10) found that the Laroche glaucoma calculator is a simple and low-cost screening tool. Using a handheld tonometer and corneal thickness gauge, a sensitivity of 93.5% and specificity of 91.3% can be achieved, with extremely high sensitivity, specificity, predictive value, and accuracy. It can be administered by trained non-physicians, making it a valuable triage tool to identify individuals who need gonioscopy confirmation of PACS, PAC, or PACG, particularly in underserved or resource-limited populations. Philip et al. (11) found that population-based data also support the use of anterior chamber depth (ACD) as a screening metric, with ACD cutoffs of ≤2.8 mm yielding 90–92% accuracy for detecting angle closure.

With the introduction of the Nd: YAG laser (12, 13), LPI has become widely used for early intervention in angle closure glaucoma. Owing to advances in laser technology and improved safety, LPI is now considered a preferred treatment for PAC and PACS and a standard approach for preventing PACG (14). Asahi (15) also demonstrated the cost-effectiveness of LPI in patients with PACS. The mechanism of LPI involves creating an opening in the peripheral iris, typically in the superior quadrant, to establish an alternative pathway for aqueous humor flow. This reduces the pressure gradient between the posterior and anterior chambers, alleviates pupillary block, flattens the convex iris configuration, widens the ACA, and increases the peripheral ACD, thereby reducing IOP.

Several clinical studies have investigated changes in anterior segment parameters before and after LPI in patients with PACS. Acet et al. (16) reported a short-term increase in central ACD after LPI in patients with PACS. By perforating the peripheral iris, LPI equalizes pressure between the anterior and posterior chambers, deepens the anterior chamber, and induces posterior displacement of the lens–iris diaphragm (17). A 5-year follow-up study from the Beijing Tongren Eye Center (18) identified baseline ACD as an independent risk factor for progression from PACS to PAC or PACG. Similarly, a randomized controlled trial from the Zhongshan Ophthalmic Center (19) demonstrated a statistically significant increase in ACA width after LPI compared with preoperative values in patients with PACS.

Recent studies have further supported the necessity of LPI in patients with PACS. Baskaran (20) conducted a self-controlled study in Singapore involving 480 patients with PACS, in which one eye of each patient underwent LPI. After 5 years of follow-up, the incidence of progression to PAC and PACG was significantly lower in treated eyes than in untreated eyes. In China, He (21) enrolled 889 patients with PACS and performed LPI in one eye of each patient; after 6 years of follow-up, the risk of progression to PAC was reduced by 47% in treated eyes. These findings indicate that LPI can reverse angle closure, rebalance pressure between the anterior and posterior chambers, deepen the ACA, stabilize IOP, and delay progression from PAC to PACG. Zhang (22) also reported that LPI effectively improves ACD and angle width and stabilizes IOP in patients with PAC. Based on these findings, LPI is recommended for patients with PACS to improve anterior segment anatomy and delay disease progression.

In the present study, gonioscopy was performed over more than 360° in patients suspected of angle closure glaucoma or with a family history of glaucoma. Patients diagnosed with PACS subsequently underwent LPI. Our results showed no reduction in visual acuity after LPI, with stable IOP and no decrease in ECD, indicating favorable safety. Liao (23) reported similar findings after a 72-month follow-up of patients with PACS treated with LPI, showing no damage to the corneal endothelium. Previous studies have demonstrated good agreement between the Sirius and Pentacam systems in measuring anterior segment parameters, such as ACD (24); therefore, the Sirius system was used in this study to evaluate ACD, ACV, and ACA. Our findings demonstrated that ACD increased, ACV enlarged, and ACA widened after LPI in patients with PACS, reflecting improvement in shallow anterior chamber and narrow-angle anatomy. These results are consistent with those reported by Tarannum (25). The occurrence of pupillary block is closely associated with the structure of the anterior chamber, with a well-structured chamber being considered conducive to a lower risk of pupillary block. Furthermore, the angle of the anterior chamber serves as the drainage pathway for the aqueous humor, and once this closes, the balance between the production of aqueous humor and outflow is disrupted, thereby inducing the onset of PACG. Consequently, on the basis of a 3-month short-term follow-up observation, it is considered that patients with PACS who underwent LPI had a reduced risk of pupillary block, which may, to some extent, lower the likelihood of PACS patients progressing to PAC or PACG. However, a 3-month follow-up period is of insufficient length to enable a valid assessment regarding the long-term efficacy of LPI. Moreover, no statistically significant differences were observed between measurements at 1 week and 3 months postoperatively, suggesting that anterior chamber structural changes stabilized within 3 months after LPI. Sahin (26) also used the Sirius system to assess anterior chamber parameters in patients with PACS and reported increases in ACV and ACA after LPI; however, ACD did not change significantly, which differs from our findings. This discrepancy may be related to their smaller sample size. Similarly, Zhang et al. (27) studied patients with acute PACG and found significant increases in ACV and ACA after LPI; in contrast, ACD remained unchanged. These differences suggest that the effect of LPI on ACD warrants further investigation with a larger sample size.

Radhakrishnan et al. (28) conducted a comprehensive evaluation of LPI efficacy in patients with PAC via a literature review. They found that approximately 2 to 57% of patients with PAC still had persistent angle closure after LPI. They concluded that LPI effectiveness is closely related to the lens characteristics. A thicker or higher lens vault would reduce LPI efficacy. In such populations, the relief effect of LPI on pupil blockage is weaker. Therefore, early removal of the lens may yield better results (29). By replacing the thick lens with a thinner artificial lens, pupil blockage can be effectively solved, ACD deepened, ACA widened, and the iris-iris meshwork contact index (ITC) reduced (30). This is cost-effective in the long term; therefore, the assessment based on the lens has become an important part of risk stratification and personalized management for high-risk populations with angle-closure glaucoma. However, in this study, owing to its short follow-up period and limited sample size, the impact of lens characteristics on LPI efficacy was not evaluated. Further inclusion of the lens characteristics in patients with PACS and analysis of the more in-depth long-term effects of LPI on the anterior chamber structure in clinical studies will be of great significance in the future.

In summary, the results reveal that LPI did not adversely affect visual acuity or ECD in patients with PACS, and IOP remained stable, which reflected the safety of the procedure. Following LPI, ACD increased, ACV enlarged, and ACA widened, with structural stability maintained for at least 3 months. We consider that performing LPI in PACS patients can improve anterior chamber anatomical structure in the short term and may delay further progression to PAC and PACG. Nevertheless, the long-term efficacy of LPI still requires further investigation.

Despite our important findings, this study does have certain limitations, notably the small sample size and the insufficiently long follow-up period of only 3 months. Accordingly, to enable an adequate assessment of whether the improvement in anterior chamber structure following LPI in PACS patients can be maintained with good long-term stability, and whether it can effectively prevent the progression from PACS to PAC or PACG, will necessitate long-term follow-up with a larger study population. Furthermore, in this study, we only analyzed changes in anterior chamber parameters before and after LPI in PACS patients without the inclusion of a suitable control group. Consequently, the observed changes in anterior chamber parameters could only be attributed to LPI. Moreover, we did not obtain longitudinal data. Thus, to facilitate a more definitive assessment of the clinical efficacy of LPI, it will be necessary to include a control group of PACS patients who did not undergo LPI treatment, analyzing changes in the structure of their anterior chambers and the proportion progressing to PAC or PACG, and comparing these changes with the rate of progression in PACS patients subjected to LPI treatment.

## Data Availability

The raw data supporting the conclusions of this article will be made available by the authors, without undue reservation.
